# Integrated Systems for NGS Data Management and Analysis: Open Issues and Available Solutions

**DOI:** 10.3389/fgene.2016.00075

**Published:** 2016-05-06

**Authors:** Valerio Bianchi, Arnaud Ceol, Alessandro G. E. Ogier, Stefano de Pretis, Eugenia Galeota, Kamal Kishore, Pranami Bora, Ottavio Croci, Stefano Campaner, Bruno Amati, Marco J. Morelli, Mattia Pelizzola

**Affiliations:** ^1^Center for Genomic Science of IIT@SEMM, Fondazione Istituto Italiano di TecnologiaMilano, Italy; ^2^Department of Experimental Oncology, European Institute of OncologyMilano, Italy

**Keywords:** high-throughput sequencing, workflow management system, genomics, epigenomics, laboratory information management system

## Abstract

Next-generation sequencing (NGS) technologies have deeply changed our understanding of cellular processes by delivering an astonishing amount of data at affordable prices; nowadays, many biology laboratories have already accumulated a large number of sequenced samples. However, managing and analyzing these data poses new challenges, which may easily be underestimated by research groups devoid of IT and quantitative skills. In this perspective, we identify five issues that should be carefully addressed by research groups approaching NGS technologies. In particular, the five key issues to be considered concern: (1) adopting a laboratory management system (LIMS) and safeguard the resulting raw data structure in downstream analyses; (2) monitoring the flow of the data and standardizing input and output directories and file names, even when multiple analysis protocols are used on the same data; (3) ensuring complete traceability of the analysis performed; (4) enabling non-experienced users to run analyses through a graphical user interface (GUI) acting as a front-end for the pipelines; (5) relying on standard metadata to annotate the datasets, and when possible using controlled vocabularies, ideally derived from biomedical ontologies. Finally, we discuss the currently available tools in the light of these issues, and we introduce HTS-flow, a new workflow management system conceived to address the concerns we raised. HTS-flow is able to retrieve information from a LIMS database, manages data analyses through a simple GUI, outputs data in standard locations and allows the complete traceability of datasets, accompanying metadata and analysis scripts.

## Introduction

Next-generation sequencing (NGS) technologies have unveiled with unprecedented detail the genomic and epigenomic patterns associated with cellular processes, therefore revolutionizing our understanding of biology. In the last years, a large number of laboratories adopted these technologies, also thanks to the steady decrease of the associated costs. However, working with NGS data inescapably creates issues in the management, storage and analysis of large and complex datasets, which are often largely underestimated: for example, a medium-sized lab (10–15 scientists) could easily generate over 500 NGS samples per year, corresponding to 1–2 terabytes of raw data.

**FIGURE 1:**
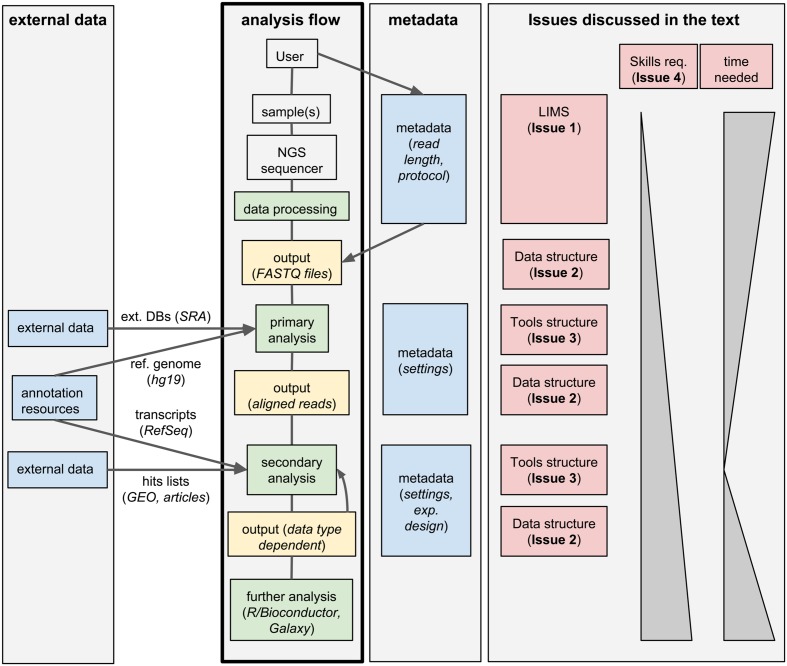
**Workflow management systems for NGS: overview and issues discussed in the text.** A typical analysis workflow for NGS is presented, associated to both the corresponding metadata and to optional additional external data. The workflow is linked to the corresponding issues discussed in the text.

Standard analysis of NGS data can be divided in two steps. First, the raw sequencing reads need to be assembled or aligned to a reference genome: this process often requires substantial computing time and infrastructures, produces large output files, but it typically does not involve much hands-on time and can be standardized for a given data type; we will call these first steps *primary analyses*. Second, biologically relevant information needs to be extracted from the assembled/aligned reads: this part of the analysis is strongly data-type-dependent, outputs small files but it may involve multiple attempts using different tools (whose parameters need to be tuned), resulting in a much larger hands-on time and potential branching of the analysis flow; we will refer to these steps as *secondary analyses*. An overview of this process is given in **Figure 1**.

We identified five issues to be addressed by labs generating and analyzing a large amount of NGS data, which we discuss below.

## Issue 1: Structuring the Raw Data

Research groups and sequencing facilities using high-throughput sequencers will quickly deal with large numbers of samples; therefore, they will likely need a laboratory information management system (LIMS) to manage the production of NGS data. A LIMS can handle information typically associated and submitted with the sequencing request. In addition, it can follow the processing of the sequencers up to the generation and archiving of the final sequencing reads. A LIMS typically relies on a database for keeping track of all the steps and includes a graphical user interface (GUI) to process user requests, as well as taking care of the maintenance of data. Specifically, a LIMS deals with general tasks such as quality controls, tracking of data, de-multiplexing of reads, and it commonly adopts a structured tree of directories for the distribution of the final data to the user (typically unaligned reads as FASTQ files).

A number of recently developed solutions are available in this field. SMITH (Venco et al., 2014) offers a complete automatic system for handling NGS data on high performance computing clusters (HPCs) and can run various downstream workflows through the Galaxy Workflow Management System (WMS) (Blankenberg et al., 2007; Boekel et al., 2015), limiting the user interaction only to administrative tasks. MendeLIMS (Grimes and Ji, 2014) is mainly focused on the management of clinical genome sequencing projects. WASP provides a basic managements system that hosts automated workflows (McLellan et al., 2012) for ChIP-Seq, RNA-Seq, miRNA-Seq, and Exome-Seq. SLIMS is a sample management tool that allows the creation of metadata information for genome-wide association studies (Van Rossum et al., 2010). The Galaxy platform offers the module Galaxy LIMS (Scholtalbers et al., 2013) providing full access to Galaxy analysis tools in addition to basic LIMS functions.

## Issue 2: Monitoring the Analysis Flow

A LIMS allows complementing raw NGS data with storage locations and metadata information, guaranteeing complete traceability, and therefore easily pinpointing inconsistencies. The subsequent analysis of those data, both at the primary and secondary levels, commonly neglects the structure provided by the LIMS, severely affecting the robustness and reproducibility of the analyses, ultimately complicating the retrieval and sharing of the final results. Indeed, renaming of files and/or their relocation in non-standard locations can often occur and ultimately impair the association of the output of the analysis to the original LIMS entries. In some cases, the lack of this link could be particularly problematic, and would obfuscate batch effects or issues affecting specific samples. For example, read length is a parameter that is usually stored in a NGS LIMS, and it is generally not conserved along the downstream analysis. Let us suppose that, during the sequencing, a sample was generated with a different read length. This sample could become an outlier due to this specific difference; yet, if the connection to this piece of information stored in the LIMS is lost, the cause of this peculiar behavior could not be easily traced. On the contrary, while the extraction of biological information from NGS data requires the creation of new and possibly complex data structures, it is essential to avoid losing the structured order given by the LIMS, allowing the traceability of the performed analyses and defining standards for reproducibility.

Importantly, the secondary data analysis often involves testing several combinations of tools and parameters, further inflating the required disk space and generating multiple outputs. Without a careful organization of the results and without tracking their association with the raw data, primary analysis, and associated metadata, very rapidly the final results could become difficult to interpret by collaborators or colleagues. Given enough time, this would likely be also the case for the person who performed the final secondary analyses. The ability to monitor the flow of NGS data from their generation to preliminary and higher-level analyses becomes then critical for dissemination and integration of the results of a single experiment in a larger community.

## Issue 3: Automatizing and Documenting Tools

Typically, a scientist with quantitative background (often a bioinformatician) is responsible for the primary and secondary analysis of NGS data. This task is accomplished through the execution of a series of existent or custom-made pipelines, which may need to be manually changed to account for the peculiarities of each given experiment. If this process is not properly managed, it could rely on copying and pasting lines of code, followed by manual renaming and moving of script files. The automation of these processes is a key point for a standardized (while flexible) analytical workflow and largely prevents the possibility of committing errors, especially for routine tasks that have to be manually repeated several times.

Automation is facilitated by the definition of modular, interconnected functions, which can be used to tailor the application of pipelines to the specific user’s needs. Modularity makes pipelines flexible, efficient, and easy to maintain and is critical when contribution from multiple people is expected; moreover, the high turnover of Ph.D. students and postdocs, implies frequent transfers of knowledge, which may result in a loss of critical information. To this regard, the usage of established versioning system (e.g., Git^1^ and Subversion^2^) allows to easily reproduce old results, to retrieve and correct errors in the code, and to increase the productivity of collaborative projects of software development. The modularity of software is also instrumental in promoting the adoption of parallel computation: given the growing field of cloud computing and multicore processors, having efficient pipelines that can distribute data and tasks across different parallel computing nodes and/or processors is a clear advantage and greatly reduces the amount of waiting time for the user.

## Issue 4: Ease of Use

In a typical scientific department or research group working with high-throughput sequencing technologies, the number of bioinformaticians in charge of the analysis of NGS data is considerably smaller than their wet-lab counterparts. This either causes an exceeding number of requests to the bioinformaticians or encourages wet-lab scientists to embark in NGS data analysis. Even in presence of consolidated and thoroughly tested pipelines, running the analysis requires being able to use command-line interfaces and some familiarity with the Linux/Unix operating systems: these skills are typically not taught in biology courses. Setting up a GUI that offers access to the pipelines would strongly increase the ease of use of the analysis framework, and disclose it to users devoid of specific training in computer science. GUI-based systems could alternatively offer the possibility of tuning the parameters of the various implemented tools, implement only default parameters, or implement automatic choices of the optimal parameters based on the input data and metadata (Pelizzola et al., 2006). In any case, the analysis should finally provide simple, standardized diagnostics and clear figures and tables summarizing standard outputs, and should possibly automatically highlight failed quality checks and inconsistencies. These last features are particularly important when inexperienced users can control the parameters of the available tools.

Equipped with these options, the analysis framework can easily be used by wet-lab scientists with minimal training (especially the part concerning secondary analyses). This would in turn free up a substantial amount of time for bioinformaticians, which could be relocated from repetitive tasks to more challenging and rewarding projects. Based on our experience, we believe this model is particularly effective for medium to large labs.

## Issue 5: Data Reproducibility

The analysis of high-throughput biological data, including NGS data, often relies on annotation data available in public databases. For example, the alignment of the reads requires the availability of FASTA files for a reference genome; the determination of absolute gene expression in RNA-seq experiments depends on having GTF files containing the structure of transcriptional units constituents (such as exons, and coding sequences). Importantly, reference genomes and other annotation data are periodically updated, and tracking their versions becomes essential to ensure compatibility with future analyses. At the same time, multiple annotation data have to be consistent with a particular reference genome build. In addition, some metadata can be retrieved by alternative providers or generated based on different criteria (see for example transcript annotations based on RefSeq or ENSEMBL). As a result, analyses based on alternative sources of metadata will likely provide different results, even if matched to the correct reference genome.

For these reasons it is fundamental to track the adopted resources and maintain compatible and updated annotation data. To this regard, projects such as Bioconductor (Gentleman et al., 2004) encourage the adoption of standard annotation packages as reference for the community of scientists working in this field, ranging from annotation databases (packages of the TxDb series) to complete genome assemblies (BSgenome packages). In this way, one could rely on those metadata packages, thus ensuring the reproducibility and comparability of the results.

Similarly, the usage of controlled vocabularies, ideally derived from biomedical ontologies, would prevent ambiguities and help properly organizing the metadata. This can help creating an unambiguous description of the type of treatment that a given cell or tissue type in a specific disease state was subjected to. The use of these resources is often encouraged, for example when publishing NGS data in large-scale repositories, and standards such as MIAME are available (Brazma et al., 2001). Nevertheless, these good practice recommendations are only sporadically applied and, as a result, querying databases of high-throughput biological data can be cumbersome. The same issue can affect the metadata contained in the LIMS and WMSs: therefore, it can be extremely useful to provide metadata specific for the primary and secondary analyses, containing a minimal description of the experimental design and the performed analysis (for examples describing the rational of comparing specific conditions within an analysis of differential expression), therefore making the results more intelligible to other scientists. Noteworthy, the availability of proper metadata for the samples and their analysis can greatly facilitate the export of the output files in public repositories.

## Workflow Management Systems

Workflow Management Systems try to cope with Issues 2–5 and are essential for efficiently managing the analysis of large NGS datasets.

Galaxy (Blankenberg et al., 2007; Boekel et al., 2015) is a popular data analysis framework that handles NGS data and allows designing articulated workflows. Its last release provides a simplified framework for integrating and/or designing new analysis pipelines, and despite being intended also for non-programmer users, it is mainly restricted to skilled bioinformaticians for complex tasks. While Galaxy addressed Issue 1 with the development of Galaxy LIMS (which supports request submissions, de-multiplexing, and delivery of the sample files), the output of the pipelines is not standardized and depends on the user. The high level of flexibility in modifying the parameters decreases a lot the automation given by this resource, limiting its usage to an audience with both a good knowledge of the tools applied and good IT skills.

Chipster (Kallio et al., 2011) is another popular WMS with a user-friendly interface that can analyze several types of NGS data (such as RNA-, miRNA-, ChIP-, and whole-genome sequencing), and save and share automatic workflows with other users. Chipster is not designed to be integrated with a LIMS: the users have to import their data manually. Workflows can be easily set up in few minutes and then repeated on several samples with little hands-on work. The lack of a LIMS-like system make this tool suitable only for laboratories with a limited amount of NGS datasets (each sample has to be loaded separately) and setting the pipelines requires a good knowledge of the tools the user is going to use.

Two recent tools, Omics Pipe (Fisch et al., 2015) and QuickNGS (Wagle et al., 2015), were developed with the main goal of making NGS analyses available to a broader audience. However, Omics Pipe is strongly oriented for IT specialists and bioinformaticians who need to analyze a large number of dataset and want to automate data analysis pipelines for multiple NGS technologies (RNA-, Exome-, miRNA-, ChIP-, and whole-genome sequencing). The Python modules at the core of Omics Pipe make it easily extendable and allow users with Unix command-line experience to execute the supported pipelines, which can be debugged and corrected thanks to the built-in version control. All these features combined make it very difficult for a biologist with average computational expertise to use Omics Pipe. On the other hand, QuickNGS allows performing most of the common operations on NGS data, such as primary analyses (filtering and alignment of the sequence reads to the reference genome) and secondary analyses (differential gene expression and differential exon usage for RNA sequencing data) with very limited prior knowledge and hands-on time. The results of the workflows are easily accessible by users through the generation of standardized spreadsheet files, plots, and web reports. The adoption of annotation databases such as BioMart or genome sequence and annotations from Ensembl, make the results highly reproducible. The web interface is extremely simple and permits to follow the operations performed on the samples, but does not allow parameter adjusting. Finally, QuickNGS does not offer integration with a LIMS and the pipelines require the samples being associated with metadata information.

Despite the availability of various WMS, a recent review highlighted that these solutions have a fundamental limit: users have to switch to multiple GUIs to execute a complete NGS analysis (Poplawski et al., 2016).

## HTS-Flow

A recent attempt to deal with the issues discussed here is the HTS-flow framework, a WMS designed for simple and efficient management and analysis of NGS data. HTS-flow is currently used in our Research Institute to cope with the analysis of several hundreds NGS samples, covering the most common data types (ChIP-seq, RNA-seq, DNase-seq, BS-seq). HTS-flow was designed to work together with a LIMS, used for the management of the NGS data from a sequencing facility. In our Research Institute, the samples submitted to sequencing are centrally managed with the SMITH LIMS (Venco et al., 2014), which takes care of keeping track and distributing the raw sequencing data to the specific research group and user. These raw NGS data are automatically visible within HTS-flow for further analysis (Issue 1). In addition, since in our experience it is often useful integrating data generated in house with public datasets and data from external research groups, we introduced the possibility to import in HTS-flow external NGS samples. For the NGS data available in the GEO and SRA repositories, HTS-flow allows to automatically import the corresponding raw data. The user has to indicate the GSM or GSE IDs of the samples interest, the corresponding SRA files are automatically downloaded and converted into FASTQ files, and features from the corresponding metadata that are critical for the analysis for the data analysis are imported. These functionalities guarantee that data coming from various sources are analyzed with identical workflows, greatly facilitating the comparison of the results.

In HTS-flow, primary analyses can be seamlessly performed as soon as the raw NGS data (FASTQ or SRA files) are tracked in the LIMS: quality controls, pre-processing, and alignment to reference genome are performed on a per-sample basis. Multiple secondary analysis solutions are available to be applied on individual samples or groups thereof. The following secondary analysis are available in HTS-flow: (i) peak calling, differential peak calling and saturation analysis for ChIP-Seq data, (ii) absolute and differential expression quantification for RNA-Seq, (iii) integrative analysis of nascent and total RNA-seq data, thus quantifying mRNA synthesis, processing and degradation rates through the INSPEcT Bioconductor package that we recently developed (de Pretis et al., 2015), (iv) identification of DHS regions and digital footprints in DNase-Seq data, (v) determination of absolute and relative methylation levels and identification of differentially methylated regions for high-throughput DNA methylation data (including both targeted and whole-genome base-resolution data).

The web interface has been designed to help users without bioinformatics skills to run primary and secondary analyses and track their progression (Issues 2 and 4). The user can choose an analysis type and directly modify the specific settings, i.e., the maximum number of mismatches allowed in the alignment process, the significance threshold for the peak caller, etc. Importantly, the results of the primary and secondary analyses can be automatically and effortlessly exported to the IGB genome-browser (Nicol et al., 2009).

HTS-flow works with a suite of predefined, easily customizable modular scripts. As NGS analyses are continuously evolving, the tools and scripts used have to be versioned and tracked for handling their evolution and hunting possible bugs (Issue 3); to account for this issue, HTS-flow was deposited to Github for versioning and distribution and the software web page can be reached at http://arnaudceol.github.io/htsflow.

HTS-flow is entirely based on standard Bioconductor metadata libraries for the annotation of transcripts and reference genomes (Issue 5) such as TxDb and BSgenome packages, while the usage of similar libraries in Galaxy, Chipster, OmicsPipe, and QuickNGS has to be implemented by expert users by modifying the available pipelines. Similarly, output data are available in HTS-flow as R data objects complying with standard Bioconductor infrastructures, to be quickly imported in R and further analyzed using R/Bioconductor packages developed for the analysis and the integration of different (epi)genomics data types, such as the compEpiTools package (Kishore et al., 2015).

The automation in HTS-flow is higher than in the other tools considered in this perspective, mainly because of the integration with the SMITH LIMS, and the (optional) possibility of tweaking parameters in the pipelines; moreover, the GUI is designed to be used by the typical wet-lab scientist. Both QuickNGS and HTS-flow allow a consistent reduction of the hands-on time users need to spend for basic NGS data analyses. In particular, with QuickNGS a user has simply to upload the sample data and wait for the completion of the analyses. However, this extremely high level of automation is achieved at the expense of flexibility, as it is not possible to set parameters. On the contrary, the GUI in HTS-flow is designed to give enough control to wet-lab scientists by choosing a few critical parameters, while leaving the rest as defaults. An overview of how several WMS handle Issues 1–5 compared with HTS-flow is given in **Table 1**.

**Table 1:** HTS-flow and other available WMSs: how they are positioned with respect to the issues discussed in the text.

	HTS-flow	Galaxy	Chipster	Omics Pipe	Quick NGS
**Issue 1** Structuring the raw data	• SMITH LIMS • database with metadata information	Galaxy LIMS (optional)	–	–	Database with metadata information
**Issue 2** Monitoring the analysis flow	via GUI and/or command line for raw data, primary and secondary	via GUI for primary and secondary analysis	via GUI for primary and secondary analysis	via command line for primary and secondary analysis	via GUI for primary and secondary analysis
**Issue 3** Automatizing and documenting tools	• Provided pipelines • user’s custom-made pipelines • version controlled	User’s custom-made pipelines	User’s custom-madepipelines	• Provided pipelines • user’s custom-made pipelines • version controlled	• Provided pipelines • user’s custom-made pipelines
**Issue 4** Ease of use	• GUI • default parameters provided • possibility to tweak some parameters	• GUI • possibility to tweak all the parameters	• GUI • default parameters provided • possibility to tweak all the parameters	• default parameters provided • possibility to tweak all the parameters	• GUI • default parameters provided
**Issue 5** Data reproducibility	Annotation based on metadata packages from TxDB, Bsgenome, compEpiTools	Annotation files provided by user	Annotation files provided by user	Annotation files provided by user	Annotation based on ENSEMBL metadata

*Recently described or very popular WMS (one for each column) are compared to HTS-flow in respect to their ability of coping with the issues described in the text (rows).*

## Perspectives

Current developments toward the automation and integration of management and analysis of sequencing data will improve the efficiency of research units heavily relying on NGS technologies. Although larger groups with enough computational members may prefer to develop and maintain their own tailored frameworks, smaller laboratories would save resources and improve their results by adopting solutions developed by third parties, so to be able to concentrate on the main scientific task, namely the interpretation of the analysis results.

One aspect that we have not covered in this manuscript is the need for substantial resources both in terms of computational power and storage. The largest institutes have usually access to large clusters and storage infrastructures, and are able to deal with the associated cost of software licenses and technologists required for maintenance; on the other hand, this hardware and resources is usually too expensive for smaller institutes. Fortunately, cloud solutions are becoming accessible: companies like Amazon^3^ and Google^4^ provide access to their computational power to store and analyze data on the cloud; these are only the most visible examples in a field in fast development. Taking advantage of this opportunity, Taverna (Oinn et al., 2004; Wolstencroft et al., 2013), another widely used WMS, can successfully process whole-genome sequencing data on the Amazon cloud. A cloud version of Galaxy and similar frameworks have also been implemented (Afgan et al., 2010, 2015).

Furthermore, solving all the issues discussed here will only be useful if the final data is released and easily accessible to the final users. HTS-flow outputs results as standard R objects, ready for further analyses with the R language; typically, wet-lab scientists are interested in visualizing them in a genome browser, such as the UCSC Genome browser (Rosenbloom et al., 2015), where genomic data can be loaded as tracks on a web interface. Next-generation browsers, often available as desktop applications, allow faster visualization and further analyses. IGB, for example, has implemented a powerful zooming, searching, and exporting functions and it can be extended to integrate the genomic results with proteomic, network and structure biology (Céol and Müller, 2015). It is therefore crucial to release the data in a format flexible enough to satisfy all users. The output could be made available preformatted as data tracks for a particular genome browser, or through standards servers implementing for instance a DAS/1 or DAS/2 service (Hubbard and Birney, 2000; Dowell et al., 2001). Although the latter has been neglected, it allows loading the data directly in several genome browsers (including IGB) and supports authentication. This last feature will be particularly important if the data are shared among several research groups.

Finally, an additional positive aspect of the integration of the data management and analyses is the possibility to leverage on the metadata associated to the samples for further analyses of these data. As the amount of genomic and epigenomic resources grows and need to be confronted with reference datasets like those from the ENCODE (The Encode Project Consortium, 2011) or the TCGA consortium (Cancer Genome Atlas Network, 2012), new querying tools as the GenoMetric Query Language (Masseroli et al., 2015) will make it possible to interrogate and compare large scale genomic information based on experimental and phenotypic properties.

## Author Contributions

VB, AC, and AO developed the HTS-flow WMS. VB, PB, SP, KK, OC, MM, and MP contributed to the development of the NGS pipelines contained in HTS-flow. VB, SC, BA, MM, and MP conceived the study, closely followed the development of HTS-flow, and participated to its testing. VB, AC, MM, and MP wrote the manuscript. All authors read and approved the final manuscript version.

## Conflict of Interest Statement

The authors declare that the research was conducted in the absence of any commercial or financial relationships that could be construed as a potential conflict of interest.

## Footnotes

1 https://git-scm.com/

2 https://subversion.apache.org/

3 https://aws.amazon.com/health/genomics/

4 https://cloud.google.com/genomics/
